# A lesson for the clinical nephrologist: desmopressin and its unforeseen efficacy in clinical post-obstructive diuresis

**DOI:** 10.1007/s40620-023-01782-x

**Published:** 2023-10-19

**Authors:** Riemer Anton Been, Philip Johannes Gerdiaan Maria Voets, Femke Christina Ching-Chuan van Rhijn-Brouwer, Nils Pieter Joost Vogtländer

**Affiliations:** 1 Department of Internal Medicine, University Medical Centre Groningen, Hanzeplein 1, 9713 GZ Groningen, The Netherlands; 2 Department of Internal Medicine, Gelre Hospital, Apeldoorn, The Netherlands; 3 Department of Internal Medicine, University Medical Centre Utrecht, Utrecht, The Netherlands

**Keywords:** Hyponatremia, Post-obstructive diuresis, Desmopressin, Overcorrection

## The case

**Fig. 1:**
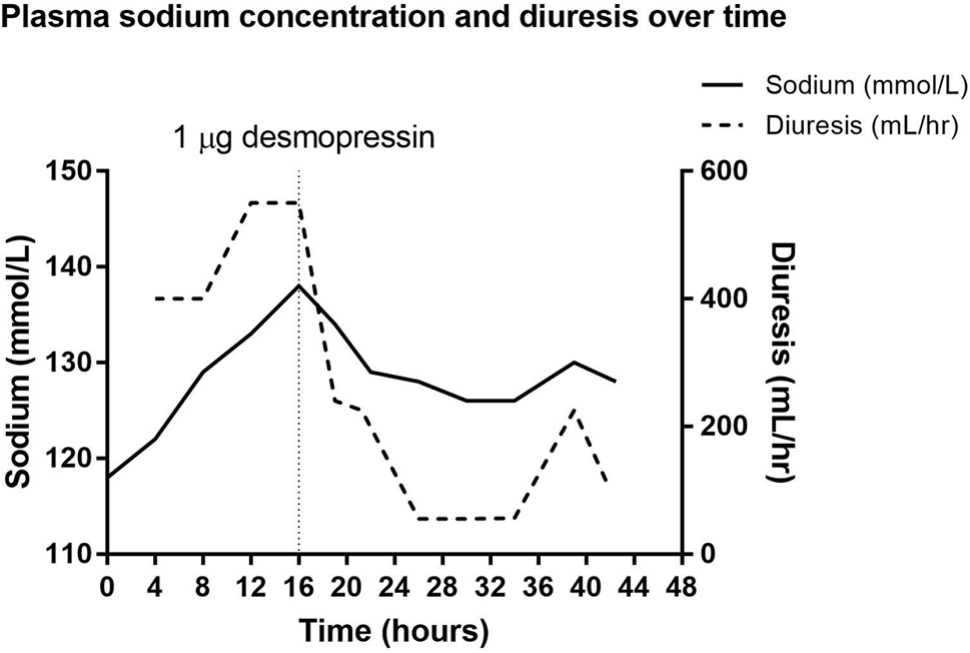
Graphical representation of plasma sodium concentrations (solid line) and diuresis (dotted line) during the first 44 h of admission, with the vertical dotted line indicating administration of desmopressin (simultaneously with high-volume fluid infusion and oral intake of water)

A 76-year-old man presented to the Emergency Care department with stranguria for five days and recurrent fever. The patient had a previous history of secondary adrenal insufficiency after pituitary surgery, treated with hydrocortisone, and of urinary retention due to benign prostate hyperplasia. Prior to presentation, the patient had doubled his hydrocortisone dosage, and one hour before admittance, the general physician had placed a catheter to alleviate urinary retention. Approximately two liters of urine were rapidly relieved. Physical examination revealed bibasal rales and pitting edema of the lower extremities. Patient was normotensive, with a heart rate of 102 beats/minute and respiratory rate of 24 breaths/minute. Laboratory tests showed elevated C-reactive protein (CRP) of 173 mg/L, a leukocytosis of 22.3/nL (neutrophils: 20.5/nL), and a plasma sodium of 118 mmol/L (osmolality: 248 mOsmol/kg), with concurrent acute renal failure (plasma creatinine: 202 µmol/L, plasma urea: 9.3 mmol/L). Urinary sodium at admission was 16 mmol/L, urine osmolality was 259 mOsmol/kg, which approximated isosthenuria due to post-renal obstruction, as there were no signs of other conditions causing isosthenuria. Inflammation parameters and fever were deemed consistent with urinary tract infection, which could have worsened the pre-existing stranguria due to prostate hypertrophy. Treatment was started with antibiotics and hydrocortisone, to prevent adrenal crisis. During admission, urine production increased, exceeding 500 mL per hour, clinically diagnosed as post-obstructive diuresis, and plasma sodium concentration increased, exceeding safe limits of 8 mmol/L/day (Fig. 1). Fluids were prescribed, with NaCl 0.9% at 250 mL/hour for 4 h. However, sodium concentrations continued to rise, and treatment was switched to NaCl 0.45%/Glucose 2.5% at 300 mL/hour from hour 8 to 12, followed by glucose 5% at 550 mL/hour from hour 12 to 16. Despite this, plasma sodium concentration continued to rise to 138 mmol/L. Desmopressin was initially considered, but dismissed, based on lack of desmopressin sensitivity in animal models of post-obstructive diuresis [1]. However, in a final effort to avoid a further rapid increase in plasma sodium levels, desmopressin 1.0 µg was administered intravenously, combined with 1L water orally and 550 mL/hour infusion of glucose 5%. Subsequently, urine production decreased to 55 mL/hour, and sodium levels decreased to 126 mmol/L, within safe limits, after which fluid infusion was discontinued. At this point, urine osmolality measured 556 mOsmol/kg, over twice the urine osmolality of 259 mOsmol/kg at admittance. At hour 39, diuresis resumed, and urine osmolality decreased to 182 mOsmol/kg, indicating loss of desmopressin effect. Afterwards, plasma sodium concentrations normalized, remaining within safe limits [2]. During admission and subsequent follow-up, no neurological sequelae were observed.

## Lesson for the clinical nephrologist

Our case shows the successful treatment of overcorrection of hyponatremia with desmopressin in a case of post-obstructive diuresis. The initial severe hyponatremia was thought to be caused by insufficient electrolyte-free water clearance (EFWC) due to post-renal obstruction, which led to water overload. The differential diagnosis of the hyponatremia was hypocortisolism or syndrome of inappropriate antidiuretic hormone (ADH) secretion due to bladder distension. In both cases, overcorrection of hyponatremia could be treated with desmopressin [3]. However, both diagnoses could be rejected. The low urinary sodium concentration, the repeated hydrocortisone boluses, and the normotensive state argued against hypocortisolism. The initial iso-osmotic urine makes excessive antidiuretic hormone secretion unlikely, as SIADH would have caused a higher urine concentration.

The subsequent overcorrection of the hyponatremia after fluid infusion was presumed to be due to post-obstructive diuresis. Alternatively, it has been suggested that severe hyponatremia may suppress endogenous ADH secretion. This can respond to desmopressin. The urine production in the first sixteen hours of admission equals the patient’s entire calculated total body water excess of 7.5–8 L, and his plasma sodium levels rose at a steady rate of almost 1.5 mmol/L/hour, despite interventions. Therefore, is seems unlikely that this was simply physiological water diuresis. In our opinion, co-existing post-obstructive diuresis seems more plausible, although accurately differentiating between these two possibilities is challenging.

Post-obstructive diuresis is defined as a diuresis > 200 mL/h for at least 2 h, or > 3000 mL/24 h after removal of an obstructive cause. It results from the renal inability to concentrate urine [4], and can cause fatal dehydration and electrolyte disturbances. The pathophysiology of post-obstructive diuresis is not completely understood. It is thought that a reduced expression of tubular transporter proteins and wash-out of the medullary osmotic gradient are involved, resulting in lessened ADH sensitivity. Ischemic damage to juxtamedullary nephrons has also been proposed [4, 5]. Desmopressin does not affect either of these mechanisms, thus desmopressin is not incorporated in treatment protocols for post-obstructive diuresis [6]. However, its utility was clearly shown in this case. As tubular ischemia is unlikely to be resolved via desmopressin (which does not affect vascular tone like vasopressin), its effects must be sought either on tubular transporter proteins, or on medullary osmotic gradient, or on a combination thereof, which we will discuss below.

Rat models have suggested that post-obstructive diuresis is caused by downregulation of tubular sodium transporters [7]. Interestingly, this downregulation should have resulted in urinary sodium loss, and consequently, in high fractional sodium excretion. However, our patient exhibited low fractional sodium excretion (0.46% at admittance), indicative of at least some remaining sodium transporter activity. Moreover, studies have demonstrated prolonged downregulation of the water channel AQP-2 in urinary tract obstruction [8], even in the presence of ADH [1]. However, as our patient responded rapidly to the administration of desmopressin, it is unlikely that de novo synthesis of AQP-2 was a principal factor, as it is a time-requiring process. Translocation of transporters seems a more likely explanation.

Another possible mechanism of action could be the enhanced restoration of the osmotic gradient. The osmotic gradient, defined as the difference in osmolality between the medullary interstitium and the collecting ducts, is indispensable for urine concentration. Adequate vascularization and urine flow are required to maintain this gradient. In urinary tract obstruction, the vascular wash-out of the medullary osmotic gradient occurs, preventing ADH-mediated water retention. Moreover, the hypotonic hyponatremia which occurs due to diminished electrolyte-free water clearance during post-renal obstruction inhibits ADH release. The lack of circulating ADH further increases electrolyte-free water loss once the urinary tract obstruction is removed. Exogenous administration of desmopressin circumvents this decreased endogenous ADH secretion. Studies have shown short-term ADH-induced stimulation of renal solute transporters, which rapidly restores medullary hypertonicity [9]. This enables the kidneys to once again concentrate urine, and decreases urine production, as observed in our case. From a physiopathologic standpoint, ADH helps to create the necessary osmotic gradient which the hormone itself uses to promote renal water retention. This effect would be more pronounced because desmopressin is often administered at supraphysiological doses compared to endogenous ADH.

Combining available knowledge on renal physiology, we hypothesize that the urinary tract obstruction resulted in loss of both kidney function and electrolyte-free water clearance, reflected by a severe water overload and hypotonic hyponatremia. After correction of the obstruction, the initial electrolyte-free water clearance led to a rapid increase in plasma sodium levels. Since the vasopressin receptors were intact, the medullary osmotic gradient was quickly restored as the urinary flow recovered. Previous research [6] shows that renal insufficiency, azotemia, high plasma sodium bicarbonate and other co-morbidities and medications are risk-factors for developing post-obstructive diuresis.

Our report suggests that in the case of clinical post-obstructive diuresis leading to rapid plasma sodium correction desmopressin could serve as an effective treatment.

## Abbreviations

CRP: C-reactive protein
EFWC: Electrolyte-free water clearance
ADH: Antidiuretic hormone
AQP-2: Aquaporin-2

## References

[1] Reyes AA, Robertson G, Klahr S (1991). Role of vasopressin in rats with bilateral ureteral obstruction. Proc Soc Exp Biol Med.

[2] Voets P, Maas R, Vogtländer NPJ, Kaasjager KAH (2022). Osmotic demyelination syndrome and thoughts on its prevention. J Nephrol.

[3] van der Bilt F, Alsma J (2023). Hyponatraemia caused by transient syndrome of inappropriate antidiuresis to urinary retention. Intern Med J.

[4] Nielsen S, Kwon TH, Dimke H, Skott M, Frøkiær J (2013). Aquaporin water channels in mammalian kidney. Seldin and Giebisch’s The Kidney: Physiol Pathophysiol.

[5] Leslie SW, Sajjad H, Sharma S. Postobstructive diuresis. Published online 2022. https://www.ncbi.nlm.nih.gov/books/NBK459387/. Accessed 25 May 2022

[6] Harrison S, Lasri A, Jabbour Y (2018). Post-obstructive diuresis: physiopathology, diagnosis and management after urological treatment of obstructive renal failure. Open J Urol.

[7] Li C, Wang W, Kwon TH, Knepper MA, Nielsen S, Frøkiaer J (2003). Altered expression of major renal Na transporters in rats with bilateral ureteral obstruction and release of obstruction. Am J Physiol Renal Physiol.

[8] Frøkiaer J, Marples D, Knepper MA, Nielsen S (1996). Bilateral ureteral obstruction downregulates expression of vasopressin-sensitive AQP-2 water channel in rat kidney. Am J Physiol.

[9] Giménez I, Forbush B (2003). Short-term stimulation of the renal Na-K-Cl cotransporter (NKCC2) by vasopressin involves phosphorylation and membrane translocation of the protein. J Biol Chem.

